# Do women reduce the gap to men in ultra-marathon running?

**DOI:** 10.1186/s40064-016-2326-y

**Published:** 2016-05-20

**Authors:** Beat Knechtle, Fabio Valeri, Pantelis T. Nikolaidis, Matthias A. Zingg, Thomas Rosemann, Christoph A. Rüst

**Affiliations:** Gesundheitszentrum St. Gallen, Vadianstrasse 26, 9001 St. Gallen, Switzerland; Institute of Primary Care, University of Zurich, Zurich, Switzerland; Department of Physical and Cultural Education, Hellenic Army Academy, Athens, Greece

**Keywords:** Sex difference, Ultra-endurance, Performance, Athlete

## Abstract

The aim of the present study was to examine sex differences across years in performance of runners in ultra-marathons lasting from 6 h to 10 days (i.e. 6, 12, 24, 48, 72, 144, and 240 h). Data of 32,187 finishers competing between 1975 and 2013 with 93,109 finishes were analysed using multiple linear regression analyses. With increasing age, the sex gap for all race durations increased. Across calendar years, the gap between women and men decreased in 6, 72, 144 and 240 h, but increased in 24 and 48 h. The men-to-women ratio differed among age groups, where a higher ratio was observed in the older age groups, and this relationship varied by distance. In all durations of ultra-marathon, the participation of women and men varied by age (p < 0.001), indicating a relatively low participation of women in the older age groups. In summary, between 1975 and 2013, women were able to reduce the gap to men for most of timed ultra-marathons and for those age groups where they had relatively high participation.

## Background

The comparison of endurance performance between sexes has been a main topic of scientific research for decades (Lepers et al. 2013; Parnell 1954; Pate and Kriska 1984). Nowadays, women compete in the same endurance and ultra-endurance sports disciplines as men. However, this was not always the case. For example, women were considered too weak to compete in running competitions in the Olympic Games well into the twentieth century (www.olympic.org). When Kathrine Switzer competed in 1967 in the ‘Boston Marathon’ as the first woman ever to run an official marathon, she pretended to be a man to be able to run in the race (www.baa.org). Although she finished the race in 4:20 h:min well ahead of many men, the organizer of ‘Boston Marathon’ tried to remove her from the race. Only in 1972, women were officially accepted to compete in the ‘Boston Marathon’ (www.baa.org).

Once women were admitted to marathon running, the comparison of sex differences in marathon running started to attract scientific interest. Whipp and Ward (1992) and Tatem et al. (2004) especially focused on running results and both predicted that women would outrun men in the future. Whipp and Ward (1992) predicted that women would outrun men in marathon running in 1998 while Tatem et al. (2004) extrapolated 100 m Olympic running sprints results from 1904 to 2004 and projected women to overtake men in 100 m sprint in the 2156 Olympic Games. Since women entered the professional running world more than half a century later than men their improvements in performance were higher than in men in the first 30 years (Tatem et al. 2004; Whipp and Ward 1992). This triggered the conclusion that women would outrun men at some point as in 1998 (Whipp and Ward 1992) or in 2156 (Tatem et al. 2004). While women were be able to reduce the sex difference in performance in the second half of the twentieth century in running from 100 m to the marathon distance (Tatem et al. 2004; Whipp and Ward 1992), Holden (2004) found a newer trend of an increasing sex difference between 1989 and 2004. In the Olympic Games, in seven out of eight disciplines in running from 100 m to the marathon distance, the mean sex difference in performance increased from 10.4 to 11.0 %, with an exception of Paula Radcliffe’s world record in marathon running in 2003 (www.iaaf.org/home). It is worth to mention that the sex difference in world records in marathon running increased since then from 8.4 % in 2003 to 9.5 % in 2013 (www.iaaf.org/home).

The occasions where women were able to beat men in long-distance running events were very rare exceptions and happened only on recreational competitions, but never on professional competitions (Knechtle et al. 2008). Therefore, it seemed that women would, if at all, outrun men first in ultra-marathons as Pamela Reed did in the 2002 and 2003 ‘Badwater’ (www.badwater.com) or Hiroko Okiyama in the 2007 ‘Deutschlandlauf’ (www.deutschlandlauf.com). Although ultra-marathons are held all over the world, official World Championships exist only for 100 km ultra-marathons (www.iaaf.org/home). Therefore, the world’s elite in ultra-running may not compete in a single race where women might outrun men. In running races up to the marathon distance, results of World Championships, Olympic Games or a World Major Series can be compared among each other. In ultra-marathons, however, race results cannot be compared because races are not standardized due to different environmental conditions such as differences in race courses and differences in changes in altitude. Generally, events over different distances with different altitude gain or differences in course profiles cannot be compared properly. Therefore, in our opinion, the best possibility to investigate trends in ultra-marathon running performance with sex difference needs to include all existing races over a certain distance or duration.

A study including the longest ultra-marathons events held up to 10 days is required to evaluate the ongoing question whether women would outrun men in ultra-marathons. In this context, the aim of the present study was to examine sex differences across time in runners of ultra-marathons varying from 6 h to 10 days with the hypothesis that women would reduce the gap to men in the last decades.

## Methods

### Ethics

All procedures used in the study were approved by the Institutional Review Board of Kanton St. Gallen, Switzerland, with a waiver of the requirement for informed consent of the participants given the fact that the study involved the analysis of publicly available data.

### Data sampling and data analysis

The data set for this study was obtained from the race website of the ‘Deutsche Ultramarathon-Vereinigung’ (DUV) (www.ultra-marathon.org). This website records all race results of all ultra-marathons held worldwide. Data of all competitors who ever participated in a 6, 12, 24, 48, 72 h, 6 days (144 h) and 10 days (240 h) ultra-marathon held worldwide between 1975 and 2013 were analysed. In time-limited races, athletes perform laps which are counted by lap counters or electronically. Any competitor is listed in the rankings as soon as she/he has completed one lap as minimum distance.

### Statistical analysis

**Fig. 1:**
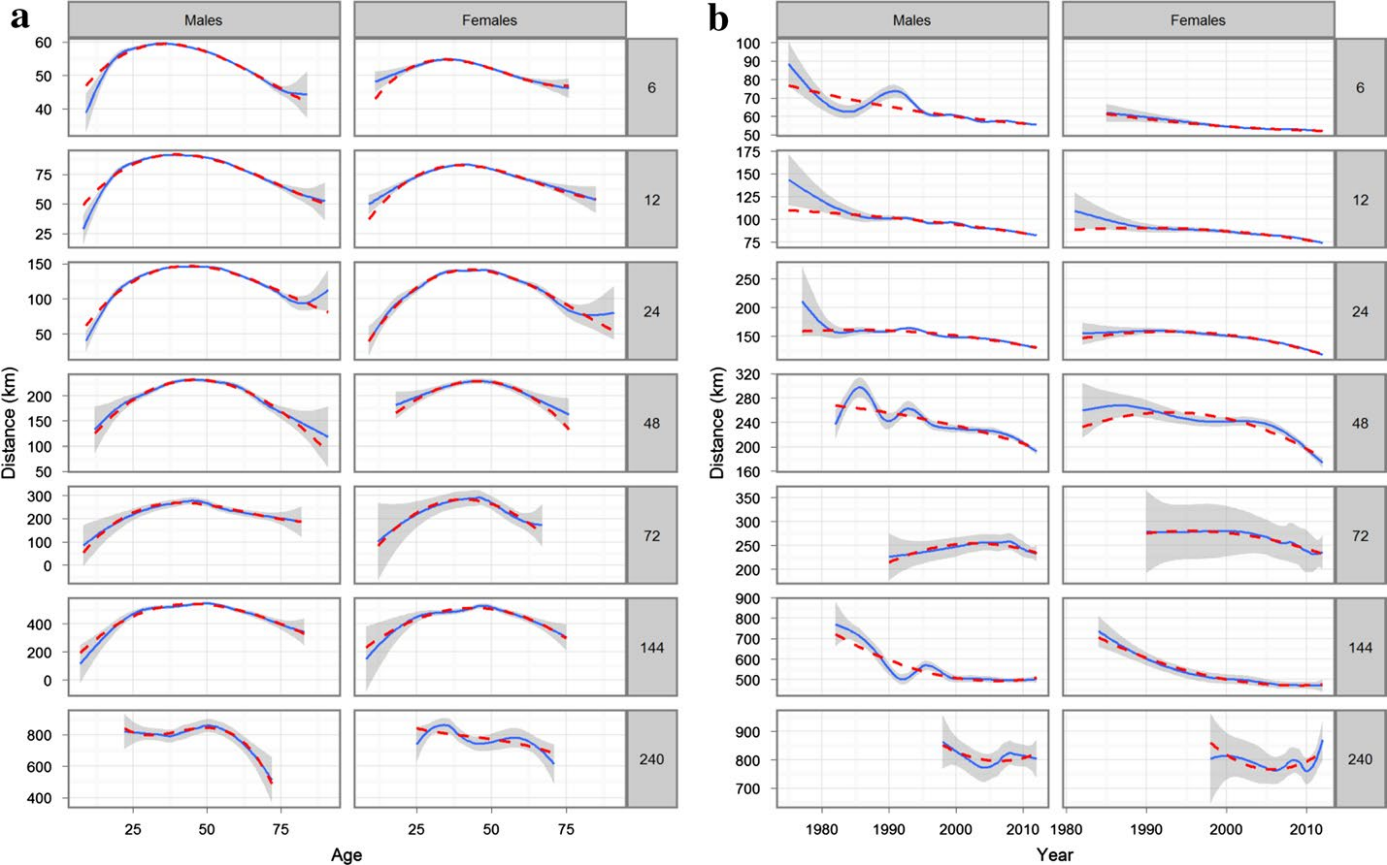
Observed distances according to age (**a**) and calendar year (**b**). *Solid line* smoothed curve. *Dashed line* cubic (**a**) and *squared* (**b**) fit

**Fig. 2:**
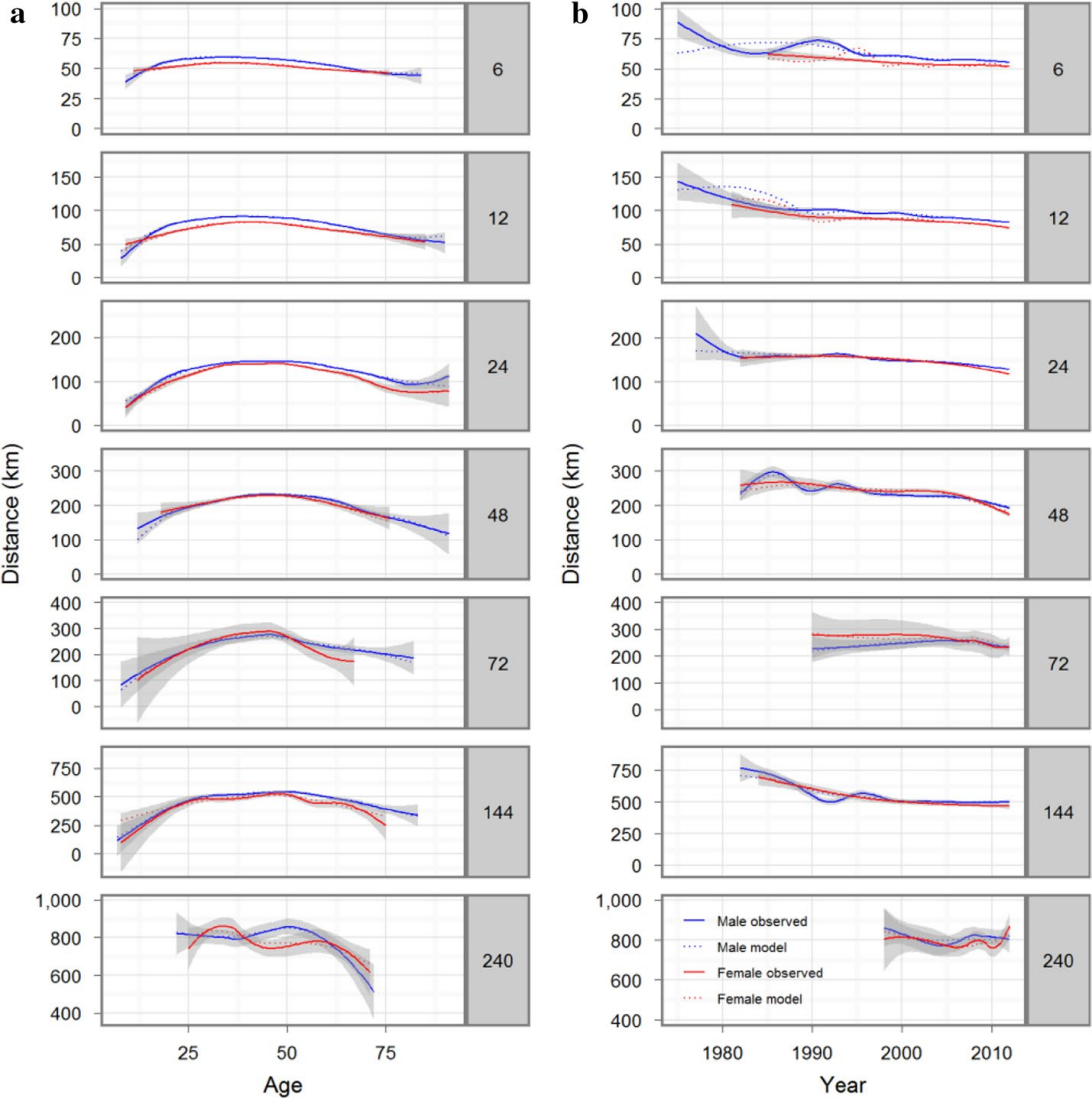
Smoothed curve of observed distances (*solid line*) and fitted distances values from model (1) (*dashed line*) according to age (**a**) and calendar year (**b**)

We used a multiple linear regression to analyse the gap between men and women (Table 1). To explore which variables may be accounted for, regression distances were graphically displayed against the variables (Fig. 1a, b). Due to the large amount of observations we used smoothing methods (i.e. loess if number of observations <1000 otherwise splines both implemented in the statistical software). The 95 % confidence regions are displayed and the polynomial fit for each ultra-marathon (UM) was added. We included the following variables in the model: sex, age at performance and calendar year of performance. To consider finishers who performed several races we included finisher as random variable in the model, although 48.9 % of the finishers in the data have only one finish. We justified including finisher as random variable by comparing the graphics of distance against age of the finisher with only one known finish with the finishers who have at least five finishes. Both graphs showed similar tendencies (graphs not shown). Visual inspection of Fig. 1a, b suggests using a cubic, quadratic and a cubic relation for age and year. To study the effect of sex we included also interactions. We also considered the heterogeneous variance of each UM-level. The final method was selected by Akaike information criterion (AIC) and visual inspection of the fitted values (Fig. 2a, b).

**Table 1:** Coefficients and standard errors from a multivariable regression model (1)

	Coefficient	Standard error	*p* value
6 h	55.7	0.13	<0.0001
Ultramarathon			
12 h	34.4	0.24	<0.0001
24 h	87.0	0.35	<0.0001
48 h	166.3	1.33	<0.0001
72 h	219.6	6.23	<0.0001
144 h	463.5	3.99	<0.0001
240 h	776.9	18.36	<0.0001
Sex (female)	−5.6	0.31	<0.0001
Age			
Age centered linear	−0.25	0.01	<0.0001
Age centered squared	−0.01	0.00	<0.0001
Age centered cube	0.00	0.00	<0.0001
Sex (female) × age			
Age centered linear	0.00	0.03	0.902
Age centered squared	0.00	0.00	0.007
Age centered cube	0.00	0.00	0.907
Year			
Year linear	−0.30	0.02	<0.0001
Year squared	0.00	0.00	0.357
Sex (female) × year			
Year linear	0.12	0.05	0.007
Year squared	−0.01	0.01	0.122
Sex (female) × ultramarathon			
12 h	−1.98	0.53	<0.0001
24 h	1.33	0.81	0.099
48 h	5.04	2.76	0.068
72 h	16.39	14.25	0.250
144 h	−19.44	8.41	0.021
240 h	−71.72	31.78	0.024
Ultramarathon 12 h × age			
Age centered linear	−0.06	0.02	0.020
Age centered squared	−0.02	0.00	<0.0001
Age centered cube	0.00	0.00	0.046
Sex (female) × ultramarathon 12 h × age			
Age centered linear	−0.02	0.05	0.678
Age centered squared	0.00	0.00	0.096
Age centered cube	0.00	0.00	0.401
Ultramarathon 24 h × age			
Age centered linear	0.19	0.04	<0.0001
Age centered squared	−0.04	0.00	<0.0001
Age centered cube	0.00	0.00	0.031
Sex (female) × ultramarathon 24 h × age			
Age centered linear	−0.13	0.09	0.131
Age centered squared	−0.01	0.00	0.002
Age centered cube	0.00	0.00	0.803
Ultramarathon 48 h × age			
Age centered linear	0.63	0.14	<0.0001
Age centered squared	−0.07	0.01	<0.0001
Age centered cube	0.00	0.00	0.749
Sex (female) × ultramarathon 48 h × age			
Age centered linear	−0.05	0.35	0.898
Age centered squared	−0.01	0.01	0.711
Age centered cube	0.00	0.00	0.484
Ultramarathon 72 h × age			
Age centered linear	−0.71	0.54	0.194
Age centered squared	−0.09	0.02	<0.0001
Age centered cube	0.00	0.00	0.033
Sex (female) × ultramarathon 72 h × age			
Age centered linear	−1.18	1.40	0.398
Age centered squared	−0.12	0.07	0.088
Age centered cube	0.00	0.00	0.717
Ultramarathon 144 h × age			
Age centered linear	0.29	0.41	0.482
Age centered squared	−0.18	0.02	<0.0001
Age centered cube	0.00	0.00	0.354
Sex (female) × ultramarathon 144 h × age			
Age centered linear	−0.10	0.99	0.923
Age centered squared	−0.01	0.04	0.727
Age centered cube	0.00	0.00	0.276
Ultramarathon 240 h × year			
Year centered linear	2.54	1.87	0.173
Year centered squared	−0.22	0.07	0.002
Year centered cube	−0.01	0.01	0.011
Sex (female) × ultramarathon 240 h × age			
Year centered linear	−4.16	3.72	0.264
Year centered squared	0.21	0.12	0.069
Year centered cube	0.01	0.01	0.420
Ultramarathon 12 h × year			
Year centered linear	−0.54	0.04	<0.0001
Year centered squared	0.00	0.00	0.324
Sex (female) × ultramarathon 12 h × year			
Year centered linear	−0.23	0.10	0.018
Year centered squared	−0.01	0.01	0.222
Ultramarathon 24 h × year			
Year centered linear	−1.32	0.07	<0.0001
Year centered squared	−0.02	0.01	<0.0001
Sex (female) × ultramarathon 24 h × year			
Year centered linear	−1.01	0.17	<0.0001
Year centered squared	−0.04	0.02	0.009
Ultramarathon 48 h × year			
Year centered linear	−2.73	0.25	<0.0001
Year centered squared	−0.04	0.02	0.016
Sex (female) × ultramarathon 48 h × year			
Year centered linear	−2.54	0.53	<0.0001
Year centered squared	−0.14	0.04	0.001
Ultramarathon 72 h × year			
Year centered linear	−1.92	1.08	0.076
Year centered squared	−0.26	0.11	0.020
Sex (female) × ultramarathon 72 h × year			
Year centered linear	−1.91	2.61	0.465
Year centered squared	0.04	0.24	0.864
Ultramarathon 144 h × year			
Year centered linear	0.75	0.84	0.372
Year centered squared	0.36	0.05	<0.0001
Sex (female) × ultramarathon 144 h × year			
Year centered linear	−1.47	1.91	0.441
Year centered squared	0.02	0.13	0.896
Ultramarathon 240 h × year			
Year centered linear	1.37	3.46	0.692
Year centered squared	0.48	0.68	0.480
Sex (female) × ultramarathon 240 h × year			
Year centered linear	5.52	5.88	0.348
Year centered squared	1.56	1.21	0.196

Variable were centered: age = 46, calendar year = 2007

The final mixed model (1) was:

1 $$ \begin{aligned} {\text{Distance}} &= {\text{sex}} \times {\text{ultra-marathon}} \times ({\text{age}} + {\text{age}}^{2} + {\text{age}}^{3} ) \\ &\quad + \,{\text{sex}} \times {\text{ultra-marathon}} \times ({\text{year}} + {\text{year}}^{2} ) \\ & \quad +\, {\text{random}}\;{\text{ID}} + {\text{weights}}\;{\text{variance}}\;{\text{UM}} \\ \end{aligned} $$

Distance is in km, ID is the identification number of the finisher, age is centered with 46 years (mean) and calendar year with 2007 (mean). Reference levels are for ultra-marathon 6 h and for sex male. To study the effect of sex according to age, years, and ultra-marathon we used estimated coefficient which has a *p* < 0.05 and computed the percentage difference of achieved km between man and women for each ultra-marathon, age and calendar year (Table 2). In addition, a Chi square test examined the variation of finishes of women and men by age group. The magnitude of the association between sex and age group was evaluated by Cramer’s V, which was interpreted as very low (less than 0.20), low (0.20–0.39), modest (0.40–0.69), high (0.70–0.89) or very high association (0.90–1.00) (Bryman and Cramer 2011). The statistical analysis and graphical outputs were performed using the statistical software R, version 3.1.2 (R Development Core Team 2008). R: A language and environment for statistical computing, R Foundation for Statistical Computing, Vienna, Austria, ISBN 3-900051-07-0, www.R-project.org.

**Table 2:** Average distances in km at reference 6 h, male sex, age 46 years, and calendar year 2007

Ultramarathon		
6 h	55.7	
12 h	90.1	
24 h	143	
48 h	222	
72 h	275	
144 h	519	
240 h	833	
Average effect of sex		
6 h	−10.0 %	
12 h	−8.4 %	
24 h	−10.0 %	n.s.
48 h	−10.0 %	n.s.
72 h	−10.0 %	n.s.
144 h	−4.8 %	
240 h	−9.3 %	

Effects in %						
Year = 2007	Age					
	36 (%)	41 (%)	46 (%)	51 (%)	56 (%)	
6 h	−6.8	−8.0	−10.0	−12.5	−15.3	
12 h	−6.8	−8.0	−10.0	−12.5	−15.3	n.s.
24 h	−2.6	−2.6	−3.0	−3.6	−4.6	
48 h	−6.8	−8.0	−10.0	−12.5	−15.3	n.s.
72 h	−6.8	−8.0	−10.0	−12.5	−15.3	n.s.
144 h	−6.8	−8.0	−10.0	−12.5	−15.3	n.s.
240 h	−6.8	−8.0	−10.0	−12.5	−15.3	n.s.

Effects in %						
Age = 46	Calendar year					
	1997 (%)	2002 (%)	2007 (%)	2012 (%)	2017 (%)	
6 h	−13.0	−11.2	−10.0	−9.5	−9.9	
12 h	−8.8	−8.0	−8.4	−10.1	−13.3	
24 h	−0.2	−0.7	−3.0	−7.4	−14.5	
48 h	3.7	3.3	−0.2	−7.9	−20.9	
72 h	−13.0	−11.2	−10.0	−9.5	−9.9	n.s.
144 h	−13.0	−11.2	−10.0	−9.5	−9.9	n.s.
240 h	−13.0	−11.2	−10.0	−9.5	−9.9	n.s.

Expressed are percentage difference between women and men. A positive percentage means that women perform better than men and vice versa. For example: women with age 36 years in 2007 performed 2.6 % less than men in ultramarathon 24 h. *n.s.* interaction effect not significant that is: gap corresponds to ultramarathon 6 h

## Results

Data of 32,187 finishers with 93,109 finishes were available. After excluding finishers with missing age, a total of 27,430 finishers and 86,508 finishes were available corresponding to a reduction of 14.8 and 7.1 %, respectively. Sex of one finisher was corrected in the case of a runner who had three runs, twice as female and one as male, assuming that she was female. Each finish should reach a minimum distance of 8, 11, 16, 22, 27, 38, and 49 km for 6, 12, 24, 48, 72, 144, and 240 h, respectively, to be included in the analysis sample, which was the case for all finishes.

**Fig. 3:**
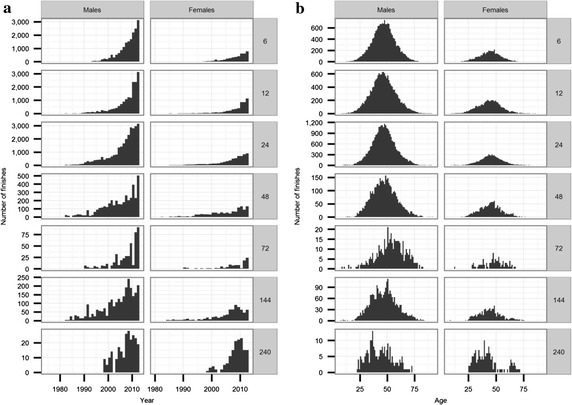
Distribution of finishes according to age (**a**), calendar year (**b**)

**Fig. 4:**
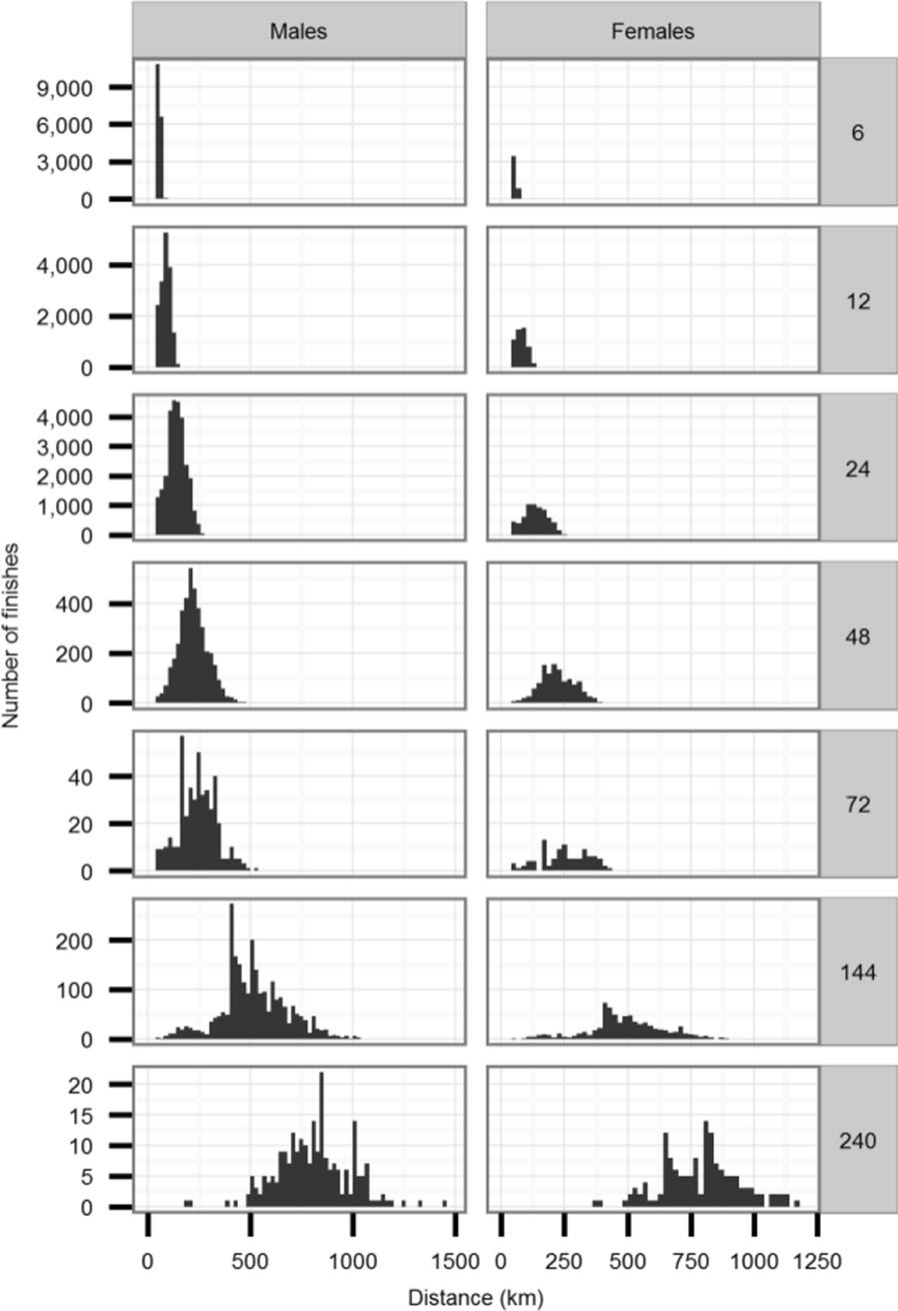
Distribution of finishes according to observed distance

Overall, 20.7 % of the finishes were performed by women and 79.3 % by men. Among all finishers, 21.7 % were women and 78.3 % were men. A total of 48.9 % of the finishers performed only one finish, 19.0 % performed two finishes and the rest of the finishers achieved three or more successful finishes during the whole period of observation (Table 3). Across calendar years, the number of finishes increased for both women and men for all events. For both women and men, most of the finishes were achieved at the age of 30–50 years (Figs. 3, 4).

**Table 3:** Distributions of number of finishes per finisher

Number of finishes	Number of finishers	Percentage of finishers (%)
1	13,409	48.9
2	5216	19.0
3	2632	9.6
4	1577	5.7
5	983	3.6
6	719	2.6
7	545	2.0
8	393	1.4
9	275	1.0
10	252	0.9
11	201	0.7
12	169	0.6
13	160	0.6
14	93	0.3
15	100	0.4
16	87	0.3
17	60	0.2
18	71	0.3
19	59	0.2
20	42	0.2
21	38	0.1
22	34	0.1
23	27	0.1
24	19	0.1
≥25	269	1.0
Total	27,430	100.0

The average distance for men in 2007 with five finishes and an age of 46 years was 55.7, 90.1, 143, 222, 275, 519, and 833 km for 6, 12, 24, 72, 144, and 240 h, respectively (Table 2). With increasing age, the sex gap for all race durations increased (Table 2, negative values mean less distance achieved than men whereas positive values mean more distance). Across calendar years, the gap between women and men decreased in 6, 72, 144 and 240 h, but increased in 24 and 48 h (Table 2). For the 1997 and 2007 calendar year at age 46 in the 48 h UM women performed better than men (+3.7 and 3.3 %).

**Fig. 5:**
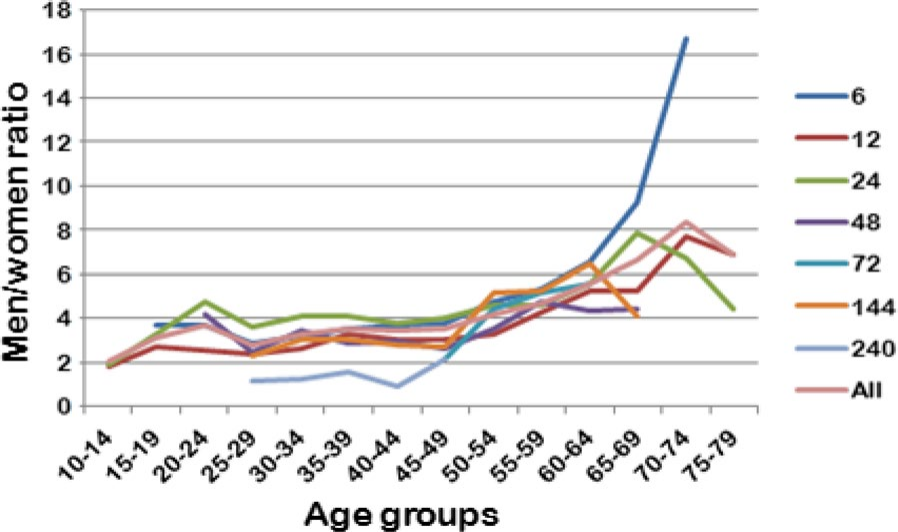
The men-to-women ratio across age groups for all race durations. Data are presented for age groups with a minimum of ten runners per sex group

The men-to-women ratio was calculated for each age group for all races (Table 4; Fig. 5). A Chi square test was performed to examine the relationship between sex and age group, i.e. whether men-to-women ratio varied by age, for each race duration. The relationship between these variables was significant for all race durations: χ^2^ = 193.2, p < 0.001, Cramer’s V = 0.09 in 6 h, χ^2^ = 166.3, p < 0.001, Cramer’s V = 0.09 in 12 h, χ^2^ = 133.8, p < 0.001, Cramer’s V = 0.06 in 24 h, χ^2^ = 61.2, p < 0.001, Cramer’s V = 0.11 in 48 h, χ^2^ = 35.1, p < 0.001, Cramer’s V = 0.26 in 72 h, χ^2^ = 68.5, p < 0.001, Cramer’s V = 0.15 in 144 h, χ^2^ = 31.7, p < 0.001, Cramer’s V = 0.29 in 240 h. According to evaluation of Cramer’s V, the magnitude of the relationship between sex and age group was very low in 6, 12, 24, 48 and 144 h and low in 72 and 240 h. That was, the men-to-women ratio differed among age groups, where a higher ratio was observed in the older age groups, and this relationship varied by distance.

**Table 4:** The number of women (W) and men (M) for each age group and race duration

Age group	6 h			12 h			24 h			48 h			72 h			144 h			240 h			Total		
	W	M	W + M	W	M	W + M	W	M	W + M	W	M	W + M	W	M	W + M	W	M	W + M	W	M	W + M	W	M	W + M
–	596	2042	2638	429	1163	1592	447	1764	2211	16	64	80	0	6	6	21	74	95				1509	5022	6622
<15	8	19	27	17	30	47	31	59	90	0	4	4	1	3	4	0	3	3				57	118	175
15–19	20	73	93	37	99	136	25	81	106	2	4	6	0	1	1	0	2	2				84	260	344
20–24	71	261	332	124	312	436	85	404	489	14	58	72	0	4	4	2	45	47	0	6	6	296	1090	1386
25–29	222	627	849	306	735	1041	259	940	1199	64	155	219	5	11	16	49	114	163	17	20	37	922	2602	3524
30–34	399	1273	1672	516	1353	1869	477	1965	2442	100	343	443	5	20	25	74	223	297	27	32	59	1598	5209	6807
35–39	630	2212	2842	659	2135	2794	862	3535	4397	174	498	672	13	25	38	108	325	433	28	43	71	2474	8773	1,1247
40–44	853	3171	4024	948	2901	3849	1342	5073	6415	221	651	872	7	45	52	147	405	552	31	28	59	3549	12,274	15,823
45–49	915	3420	4335	972	2971	3943	1369	5507	6876	265	696	961	24	51	75	149	401	550	16	34	50	3710	13,080	16,790
50–54	617	2916	3533	719	2369	3088	947	4345	5292	168	597	765	18	80	98	78	400	478	3	29	32	2550	10,736	13,286
55–59	331	1755	2086	383	1615	1998	576	2655	3231	91	429	520	13	67	80	46	240	286	2	14	16	1442	6775	8217
60–64	157	1031	1188	176	925	1101	276	1543	1819	56	245	301	10	56	66	25	162	187	10	17	27	710	3979	4689
65–69	56	518	574	101	528	629	108	853	961	31	138	169	2	36	38	21	86	107	9	5	14	328	2164	2492
70–74	11	184	195	35	268	303	69	465	534	6	78	84	0	31	31	8	64	72	2	3	5	131	1093	1224
75–79	5	50	55	14	96	110	24	107	131	1	28	29	0	11	11	1	18	19				45	310	355
80–84	0	6	6	2	26	28	3	32	35	0	13	13	0	2	2	0	8	8				5	87	92
85–89				1	7	8	1	14	15	0	2	2										2	23	25
90–94				0	2	2	4	4	8	0	1	1										4	7	11
Total	4891	19,558	24,449	5439	17,535	22,974	6905	29,346	36,251	1209	4004	5213	98	449	547	729	2570	3299	145	231	376	19,416	73,693	93,109

## Discussion

This study intended to examine the sex difference for ultra-marathons held from 6 h to 10 days with the hypothesis that women would reduce the gap to men in the last decades. The most important findings were that (1) men were faster than women for all race durations, (2) the sex gap for all race durations increased with increasing age and (3) the gap between women and men decreased in 6, 72, 144 and 240 h, but increased in 24 and 48 h between 1975 and 2013.

### Women were not able to narrow the gap to men with increasing race duration

A first important finding was that men were faster than women. The differences between women and men were between 0.2 and 10.0 % for all durations for calendar year 2007 (Table 2). However, these differences were lower than the general sex difference of 11–12 % reported for endurance and ultra-endurance performance (Cheuvront et al. 2005; Coast et al. 2004; Lepers and Cattagni 2012).

An approach of supporting the assumption of women outrunning men was reported by Speechly et al. (1996) comparing performances of both sexes in 90 km events, while matching marathon times of female and male runners. They found that women performed better than men in a 90 km event. Addressing the assumption of Speechly et al. (1996), Hoffman (2008) matched both sexes for running times in 50, 80 and 161 km in the same year and found no difference in running speed. It is important to mention that the runners investigated by Speechly et al. (1996) and Hoffman (2008) were matched for the running speed in shorter races. Therefore, the conclusion that women were as fast as men in ultra-marathon running is only partly true as no women exist who can be matched with the fastest men in the shorter running distances.

Sex differences in running performance have been shown to vary by race’s distance. For instance, Cheuvront et al. (2005) reported a sex difference of 8–14 % for running distances from 1500 m to 42 km, Lepers and Cattagni (2012) a sex difference of ~11 % in the ‘New York City Marathon’ from 1980 to 2009 and Coast et al. (2004) a sex difference of ~12.4 % in running distances from 100 m to 200 km. Across all these distances the sex difference in performance seemed rather to increase than to decrease with increasing race distance. The 240 h races belong to the longest races held worldwide (www.ultra-marathon.org) and therefore serve well for the statement that women will not outrun men in ultra-running distances.

The most important differences between women and men regarding running performance are differences in physiology and anthropometry. Women have more body fat than men in both elite (Vernillo et al. 2013) and recreational (Hoffman et al. 2010a, b) athletes. In elite runners both sexes are considerably leaner than recreational runners (Hetland et al. 1999). In both elite and recreational runners the percentage of body fat is higher in women compared to men (Blaak 2001). It could be argued that fatty tissue may be used as an energy reserve and this could be an advantage for ultra-distances since runners tend to lose body fat during multi-hours running competitions (Karstoft et al. 2013; Schütz et al. 2013). Women might benefit from their higher percentage of body fat since both sexes lose a similar amount of fat during an ultra-endurance performance such as a 100 km ultra-marathon (Knechtle et al. 2010a, b, 2012a, b). Another sex difference in anthropometry is the percentage of skeletal muscle mass (Holden 2004). In ultra-marathoners, both sexes have a lower body fat percentage and the percentage of skeletal muscle tissue is higher (Knechtle et al. 2010a, b, 2012a, b) than in recreational runners. However, body fat and training characteristics, not skeletal muscle mass, were associated with running times in half-marathoners, marathoners, and ultra-marathoners (Knechtle et al. 2012a, b).

Considering physiological aspects, maximum oxygen uptake (VO_2_max) was considered as the most significant predictor of athletic performance (Bassett and Howley 2000). While elite male athletes reach a VO_2_max of ~85 ml min^−1^ kg^−1^ (Saltin and Astrand 1967), VO_2_max is lower in elite women with a maximum of ~70 ml min^−1^ kg^−1^ (Ridout et al. 2010). VO_2_max is mainly dependent from the heart’s performance and the lung capacity (Steding et al. 2010). The maximal cardiac output (Fomin et al. 2012) and the maximal lung capacity (Guenette et al. 2007) are higher in elite male compared to elite female athletes. VO_2_max depends directly from both maximal cardiac output and lung capacity and is therefore larger in men than in women (Steding et al. 2010).

Another important aspect for running performance is running economy (Anderson 1996; Piacentini et al. 2013). Running economy is defined as the necessary effort to transport 1 kg of weight for 1 m (Morgan et al. 1989). Although there is a significant difference in running economy between elite and recreational runners, sexes show no difference (Morgan et al. 1989). Bassett and Howley (2000) found VO_2_max, body fat and running economy as the major three factors contributing and predicting running performance. Therefore, women are disadvantaged in two out of three factors and have no chance to outrun men.

### The sex gap for all race durations increased with increasing age

A second important finding was that the sex differences in performance were larger in the older runners. This discrepancy among age groups should be attributed to the men-to-women ratio in each age group. This ratio increased consistently with increasing age for most of the race durations, i.e. a relatively lower number of women participated in the older age groups compared to men. An increase in sex difference in age group athletes has already been reported for athletes competing in shorter race distances. For age group pool swimmers and marathon runners, the sex difference increased with age. However, the increase in sex difference was lower in running compared to swimming (Senefeld et al. 2016). The increase in sex difference in these ultra-marathoners was due to the lower number of women in older age groups. This finding has already been reported for runners in short distances. For marathoners, the increase in sex difference with increasing age was explained by the lower number of women compared to men (Hunter and Stevens 2013).

### The gap between women and men across calendar years

A third important finding was that the gap between the sexes decreased for certain ultra-marathons (i.e. 6, 72, 144 and 240 h) across years but increased for others (i.e. 24 and 48 h). Findings for a decrease in sex difference were reported over a large variety of distances as in 100 m sprints (Tatem et al. 2004), marathons (Whipp and Ward 1992) and ultra-marathons (Da Fonseca-Engelhardt et al. 2013; Eichenberger et al. 2012). Promoters of the theory that women would outrun men favoured linear models in performance to support their theory (Tatem et al. 2004; Whipp and Ward 1992). The use of linear models was, however, controversially discussed (Reinboud 2004) but mainly found to be worse than non-linear models.

A potential explanation for the increase in sex difference could be the participation in ultra-marathons. Several studies reported an increase in the percentage of female ultra-marathoners (Da Fonseca-Engelhardt et al. 2013; Hoffman et al. 2010a, b; Zingg et al. 2013). The first women officially ran a marathon in 1967 (www.baa.org). Forty-six years ago, the percentage of female overall finishers started to increase at the ‘Boston Marathon’ from less than 1 to 39.5 % (www.baa.org) as well as in other marathons (www.worldmarathonmajors.com). In ultra-marathons such as the ‘Western State 100 Mile Endurance Run’, the percentage of female finishers increased from virtually none in the late 1970s to nearly 20 % since 2004 (Hoffman et al. 2010a, b). Therefore, the density of both elite and recreational female finishers increased. Nevertheless, the density of the world’s fastest runners is still lower in women than in men (Deaner 2013). This leaves a possibility of a further decrease in the sex difference in ultra-running performance in case the number of female finishers will match with the number of male finishers.

The change in sex difference in performance differed between different distances (Bam et al. 1997; Tatem et al. 2004; Whipp and Ward 1992) as well as between elite and recreational runners (Hunter et al. 2011). As short and middle distance races up to 10,000 m have been held longer for both sexes in the Olympic Games, the first Olympic marathon for women was held in 1984 (www.olympic.org). Even later, women started to compete in ultra-marathons (www.ultra-marathon.org; Hoffman et al. 2010a, b). Therefore, the improvement of female performance would be faster in the first years than the improvement in men. While the sex difference in performance stabilized in running distances up to the marathon distance (Hunter et al. 2011), the sex difference in performance still decreased in ultra-marathons (Hoffman et al. 2010a, b).

### Strength, weakness, limitations and implications for future research

The strength of the study is the inclusion of all athletes competing in ultra-marathons in duration between 6 h and 10 days. Furthermore, multiple finishes per athlete were included since the aspect of previous experience seems very important in ultra-marathon running (Hoffman and Parise 2015; Knechtle et al. 2009, 2011a, b). To the best of our knowledge, the data set is the most extensive for ultra-running in time-limited ultra-marathons so far. A possible weakness could be that some events from 6 h to 10 days were not recorded in the data base and therefore were not included in the data set. Furthermore, the study is limited since variables such as anthropometric characteristics (Knechtle et al. 2009, 2010a, b, 2011a, b), training data (Hagan et al. 1981), nutrition (Maughan and Shirreffs 2012; Rodriguez et al. 2009), fluid intake (Williams et al. 2012), exercise-associated hyponatremia (Hoffman et al. 2013), physiological parameters (Billat et al. 2001), and environmental conditions (Ely et al. 2007) were not considered. These variables may have had an influence on race outcome. Future studies may investigate the sex difference for all running distances from 60 m to 3100 miles for the world fastest women and men.

### Practical applications

Despite these limitations, the findings of the present study would have important practical implications for both researchers and practitioners working with long-distance runners. Since the analysed data were the most extensive ever studied in time-limited ultra-marathons and covered a large period (~40 years), the findings might be used in future studies as reference. Moreover, runners and practitioners working with them (e.g. fitness trainers) should consider the identified sex differences in the present study in order to develop sex-tailored training programs.

## Conclusions

In time-limited races held during the 1975–2013 period, men were faster than women for all race durations, the sex gap for all race durations increased with increasing age and the sex gap decreased in 6, 72, 144 and 240 h, but increased in 24 and 48 h. Female ultra-marathoners seemed to be able to narrow the gap to men in some ultra-marathon race durations in the last 40 years. The men-to-women ratio differed among age groups, where a higher ratio was observed in the older age groups, and this relationship varied by distance.

## References

[CR1] Anderson T (1996). Biomechanics and running economy. Sports Med.

[CR2] Bam J, Noakes TD, Juritz J, Dennis SC (1997). Could women outrun men in ultramarathon races?. Med Sci Sports Exerc.

[CR3] Bassett DR, Howley ET (2000). Limiting factors for maximum oxygen uptake and determinants of endurance performance. Med Sci Sports Exerc.

[CR4] Billat VL, Demarle A, Slawinski J, Paiva M, Koralsztein JP (2001). Physical and training characteristics of top-class marathon runners. Med Sci Sports Exerc.

[CR5] Blaak E (2001). Gender differences in fat metabolism. Cur Opin Clin Nutr Metabol Care.

[CR6] Bryman A, Cramer D (2011). Quantitative data analysis with IBM SPSS 17, 18 & 19—a guide for social scientists east sussex.

[CR7] Cheuvront SN, Carter Iii R, Deruisseau KC, Moffatt RJ (2005). Running performance differences between men and women: an update. Sports Med.

[CR8] Coast JR, Blevins JS, Wilson BA (2004). Do gender differences in running performance disappear with distance?. Can J Appl Physiol.

[CR9] Da Fonseca-Engelhardt K, Knechtle B, Rüst CA, Knechtle P, Lepers R, Rosemann T (2013). Participation and performance trends in ultra-endurance running races under extreme conditions—’Spartathlon’ versus ‘Badwater’. Extrem Physiol Med.

[CR10] Deaner RO (2013). Distance running as an ideal domain for showing a sex difference in competitiveness. Arch Sex Behav.

[CR11] Eichenberger E, Knechtle B, Rüst CA, Rosemann T, Lepers R (2012). Age and sex interactions in mountain ultramarathon running—the Swiss Alpine Marathon. Open Access J Sports Med.

[CR12] Ely MR, Cheuvront SN, Roberts WO, Montain SJ (2007). Impact of weather on marathon-running performance. Med Sci Sports Exerc.

[CR13] Fomin Å, Ahlstrand M, Schill HG, Lund LH, Ståhlberg M, Manouras A, Gabrielsen A (2012). Sex differences in response to maximal exercise stress test in trained adolescents. BMC Pediatr.

[CR14] Guenette JA, Witt JD, McKenzie DC, Road JD, Sheel AW (2007). Respiratory mechanics during exercise in endurance-trained men and women. J Physiol.

[CR15] Hagan RD, Smith MG, Gettman LR (1981). Marathon performance in relation to maximal aerobic power and training indices. Med Sci Sports Exerc.

[CR16] Hetland ML, Haarbo J, Christiansen C (1999). Regional body composition determined by dual-energy X-ray absorptiometry relation to training, sex hormones, and serum lipids in male long-distance runners. Scand J Med Sci Sports.

[CR17] Hoffman MD (2008). Ultramarathon trail running comparison of performance-matched men and women. Med Sci Sports Exerc.

[CR18] Hoffman MD, Parise CA (2015). Longitudinal assessment of the effect of age and experience on performance in 161-km ultramarathons. Int J Sports Physiol Perform.

[CR19] Hoffman MD, Lebus DK, Ganong AC, Casazza GA, Loan MV (2010). Body composition of 161-km ultramarathoners. Int J Sports Med.

[CR20] Hoffman MD, Ong JC, Wang G (2010). Historical analysis of participation in 161 km ultramarathons in North America. Int J Hist Sport.

[CR21] Hoffman MD, Fogard K, Winger J, Hew-Butler T, Stuempfle KJ (2013). Characteristics of 161-km ultramarathon finishers developing exercise-associated hyponatremia. Res Sports Med.

[CR22] Holden C (2004). An everlasting gender gap?. Science.

[CR23] Hunter SK, Stevens AA (2013). Sex differences in marathon running with advanced age: physiology or participation?. Med Sci Sports Exerc.

[CR24] Hunter SK, Stevens AA, Magennis K, Skelton KW, Fauth M (2011). Is there a sex difference in the age of elite marathon runners?. Med Sci Sports Exerc.

[CR25] Karstoft K, Solomon TP, Laye MJ, Pedersen BK (2013). Daily marathon running for a week-the biochemical and body compositional effects of participation. J Strength Cond Res.

[CR26] Knechtle B, Duff B, Schulze I, Kohler G (2008). The effects of running 1,200 km within 17 days on body composition in a female ultrarunner—Deutschlandlauf 2007. Res Sports Med.

[CR27] Knechtle B, Wirth A, Knechtle P, Zimmermann K, Kohler G (2009). Personal best marathon performance is associated with performance in a 24-h run and not anthropometry or training volume. Br J Sports Med.

[CR28] Knechtle B, Rosemann T, Knechtle P, Lepers R (2010). Predictor variables for a 100-km race time in male ultra-marathoners. Percept Motor Skills.

[CR29] Knechtle B, Senn O, Imoberdorf R, Joleska I, Wirth A, Knechtle P, Rosemann T (2010). Maintained total body water content and serum sodium concentrations despite body mass loss in female ultra-runners drinking ad libitum during a 100 km race. Asia Pacif J Clin Nutr.

[CR30] Knechtle B, Knechtle P, Rosemann T, Lepers R (2011). Personal best marathon time and longest training run, not anthropometry, predict performance in recreational 24-hour ultrarunners. J Strength Cond Res.

[CR31] Knechtle B, Knechtle P, Rosemann T, Senn O (2011). What is associated with race performance in male 100-km ultra-marathoners anthropometry, training or marathon best time?. J Sports Sci.

[CR32] Knechtle B, Knechtle P, Wirth A, Rüst CA, Rosemann T (2012). A faster running speed is associated with a greater body weight loss in 100-km ultra-marathoners. J Sports Sci.

[CR33] Knechtle B, Rüst CA, Knechtle P, Rosemann T (2012). Does muscle mass affect running times in male long-distance master runners?. Asian J Sports Med.

[CR34] Lepers R, Cattagni T (2012). Do older athletes reach limits in their performance during marathon running?. Age.

[CR35] Lepers R, Knechtle B, Stapley PJ (2013). Trends in triathlon performance: effects of sex and age. Sports Med.

[CR36] Maughan RJ, Shirreffs SM (2012). Nutrition for sports performance: issues and opportunities. Proc Nutr Soc.

[CR37] Morgan DW, Martin PE, Krahenbuhl GS (1989). Factors affecting running economy. Sports Med.

[CR38] Parnell RW (1954). The relationship of masculine and feminine physical traits to academic and athletic performance. Br J Med Psychol.

[CR39] Pate RR, Kriska A (1984). Physiological basis of the sex difference in cardiorespiratory endurance sports medicine. Int J Appl Med Sci Sport Exerc.

[CR40] Piacentini MF, De Ioannon G, Comotto S, Spedicato A, Vernillo G, La Torre A (2013). Concurrent strength and endurance training effects on running economy in master endurance runners. J Strength Cond Res.

[CR41] Reinboud W (2004). Linear models can’t keep up with sport gender gap. Nature.

[CR42] Ridout SJ, Parker BA, Smithmyer SL, Gonzales JU, Beck KC, Proctor DN (2010). Age and sex influence the balance between maximal cardiac output and peripheral vascular reserve. J Appl Physiol.

[CR43] Rodriguez NR, Di Marco NM, Langley S (2009). American College of Sports Medicine position stand. Nutrition and athletic performance. Med Sci Sports Exerc.

[CR44] Saltin B, Astrand PO (1967). Maximal oxygen uptake in athletes. J Appl Physiol.

[CR45] Schütz UHW, Billich C, König K, Würslin C, Wiedelbach H, Brambs HJ, Machann J (2013). Characteristics, changes and influence of body composition during a 4486 km transcontinental ultramarathon: results from the Transeurope Footrace mobile whole body MRI-project. BMC Med.

[CR46] Senefeld J, Joyner MJ, Stevens A, Hunter SK (2016). Sex differences in elite swimming with advanced age are less than marathon running. Scand J Med Sci Sports.

[CR47] Speechly DP, Taylor SR, Rogers GG (1996). Differences in ultra-endurance exercise in performance-matched male and female runners. Med Sci Sports Exerc.

[CR48] Steding K, Engblom H, Buhre T, Carlsson M, Mosén H, Wohlfart B, Arheden H (2010). Relation between cardiac dimensions and peak oxygen uptake. J Cardiovasc Magnet Reson.

[CR49] Tatem AJ, Guerra CA, Atkinson PM, Hay SI (2004). Momentous sprint at the 2156 Olympics?. Nature.

[CR50] Vernillo G, Schena F, Berardelli C, Rosa G, Galvani C, Maggioni M, La Torre A (2013). Anthropometric characteristics of top-class Kenyan marathon runners. J Sports Med Phys Fitness.

[CR51] Whipp BJ, Ward SA (1992). Will women soon outrun men?. Nature.

[CR52] Williams J, Tzortziou-Brown V, Malliaras P, Perry M, Kipps C (2012). Hydration strategies of runners in the London marathon. Clin J Sport Med.

[CR53] Zingg MA, Knechtle B, Rüst CA, Rosemann T, Lepers R (2013). Analysis of participation and performance in athletes by age group in ultramarathons of more than 200 km in length. Int J Gen Med.

